# Supplementary material for: Personal and relational changes following deep brain stimulation for treatment-resistant depression: A prospective qualitative study with patients and caregivers
Source: PLoS One. 2023 Apr 6;18(4):e0284160. doi: 10.1371/journal.pone.0284160 (PMC10079124; doi:10.1371/journal.pone.0284160)
Supplement: S1 File — (DOCX) [file pone.0284160.s001.docx]

**Supplementary Material**

**PATIENT INTERVIEW SCHEDULE – PRE-SURGERY**

**1. Background on DBS**

We are going to start with a brief discussion about deep brain stimulation:

- Do you remember when you first learnt about DBS? Can you tell me about it?
- Have you done any personal research?
  - *Prompt*: e.g. newspapers, online, websites, blogs, spoken to others

**2. Expectations and perspectives**

- What changes would you like to see as a result of DBS? What do they want from the procedure?
- How do you picture yourself a year after surgery?
- In which areas do you hope to experience changes?
- In which areas do you **not** expect changes?
- What are your thoughts on the extent of the benefits DBS could provide?
- After DBS, how much work do you anticipate you will have to contribute towards your recovery?
  - *Prompt*: Amount of psychological/therapeutic work, other medical interventions, support services?

**3. Personality and identity**

We are now going ask some questions about the impact DBS might have upon you as a person:

- Do you think DBS could change who you are? Or change your personality? If *yes*, how? If *no*, is it something you’ve thought about?
  - *Prompt*: be like the person you were before you were ill? / be a new and different person? / be more or less the same?
- How long do you expect these changes to last?
- How do you feel about the prospect of having the stimulator as a part of you and your body?
- Do you anticipate it will change how you see yourself? How?

**4. Inter-personal relationships**

I now want to ask you about the impact DBS might have upon your relationships with others:

- Do you think your relationship with (caregiver’s name) will change after DBS? If *yes*, how might it look like?
- In what ways would you like your relationship to change?
- Do you have any concerns about how your relationship may change after DBS?
- Do you have any concerns about how your relationship may **not** change?
- Do you think your relationships with other family and friends will change after DBS? If *yes*, how?

**5.** **Informed consent and decision-making**

We are now going to discuss the decision to undergo DBS:

- Can you describe the process of making this decision? For instance, did you go through stages of changing your mind?
- When you were making the decision to undergo DBS, whose opinion or what information was most influential or important to you?
- What risks or side effects *related to the surgery* are you aware of?
- What concerns do you have about the surgery?
- After surgery, what risks or side effects *related to the stimulation* are you aware of? What worries do you have about the stimulation?
- How informed do you feel you about the risks and side effects of DBS? In the short term (i.e. in undergoing surgery)? In the long-term?

**6. Stimulator management**

After the surgery, managing and controlling the stimulator becomes an important issue. Your stimulator settings are going to be adjusted throughout the trial.

- What changes do you expect could occur when these adjustments happen?
- How long do you think it will take for the changes to happen?
- Do you have any concerns about the stimulation adjustments and what changes might occur?
- Do you feel prepare to manage it?
- In terms of managing the stimulator yourself, have you thought about any issues you might face?
  - *Prompt*: recharging, travelling with stimulator

We’re going to wrap up soon, I just have one final questions:

- Are there things that you would like to do or are looking forward to doing after DBS?
- *Prompt*: socialising, leisure activities/hobbies, work/volunteering/study

**I’m just going to take a moment to look through my questions and make sure there are no points we’ve missed, while I’m doing this do you want to take a moment and think whether there are any issues important to you that we haven’t discussed?**

**PATIENT INTERVIEW SCHEDULE – POST-SURGERY**

**1. Background on DBS**

We met several months ago. We would like to better understand your experience since receiving DBS.

- Can you explain what has happened since then? Has anything changed in your life? If so, please describe?
- Can you briefly comment on the experience of having the DBS surgery itself?

**2. Experiences and perspectives**

- Do you recall what your original expectations regarding DBS? Were they met? (e.g., in terms of life activities and specific symptoms)?
- Have you seen any improvements?
- Are there areas you have not seen improvements that you expected to?

**3. Personality and identity**

- If at all, how has DBS changed who you are or your personality?
- Do you feel like you are a different person in anyway now?
- Do you see yourself differently since DBS? In what way? When did these changes start to occur?
- Have your relatives and/or close friends noticed changes in you? What kind of comments or observations have they made?
- What do you think brought on these changes (the device or feeling better)?
- How have you adjusted to having the device as part of you and your body?

**4. Inter-personal relationships**

- How has (caregiver) reacted to the DBS?
- Do others see you differently since the DBS?
- How would you describe your relationships with others since DBS?
- *Prompt:* better, easier, strained, more complex?
- Are there tensions or conflicts between you and others that have arisen since DBS? Or that have resolved?
- Who do you seek advice from regarding any issues like these? Do you ever discuss these sorts of issues with your team at follow-up appointments?

**5. Informed consent and decision-making**

- Knowing what you know now, if you have your time again would you undergo DBS?
- How informed about the risks and side effects of surgery do you think you were? Do you think you fully appreciated these?
- Are there things that have come as a surprise to you?
- Looking back, are there things you would have liked to have known?

**6. Device control issues**

- Who controls your stimulator being on/off or change the parameters? Has it brought about any challenges?
- Who do you contact if you have concerns about your stimulator?

**7. Public understanding and knowledge transfer**

- Are there things that the general public should know about DBS for depression?
- Have you or would you recommend this procedure to other patients/caregivers?

**Is there anything that we haven’t talked about that you would like to?**

**CAREGIVER interview SCHEDULE – Pre-Surgery**

**1. Background on MDD, DBS**

We are going to start with a brief discussion about ________’s depression and how the option of DBS came about:

- Can you briefly tell me about your relationship with _________ and the treatments they have tried for depression thus far?
- What has changed for you in the way you live since _________ developed depression?
- Do you remember when you first learnt about DBS? Can you tell me about it?
- How was the option of DBS proposed to ________?
- Can you describe the process of making this decision? For instance, did you and/or _________ go through stages of changing your minds?
- When you were making the decision to undergo DBS, whose opinion or what information was most influential or important to you?
- Have you done any personal research?
  - *Prompt*: e.g. newspapers, online, websites, blogs, spoken to others

**2. Expectations and perspectives**

- What changes would you like to see as a result of DBS? What do you want the procedure to do for _________?
- How do you picture________ a year after surgery?
- How do picture yourself a year after surgery?
- In which areas do you hope ________ will experience changes?
- In which areas do you **not** expect ________ to experience changes?
- What are your thoughts on the extent of the benefits DBS could provide?
- After DBS, how much work do you anticipate _______ will have to contribute towards their recovery?
  - *Prompt*: Amount of psychological/therapeutic work, other medical interventions, support services?
- We know that some people can hold high expectations for DBS, what about ________’s expectations?

**3.** **Personality and identity**

I want to shift focus now and ask some questions about the impact DBS might have upon ________ as a person:

- Do you think DBS could change who ________ is? Or change their personality in some way?
  - If *yes*, how? If *no*, is it something you’ve thought about?
  - *Prompt*: become like the person they were before they were ill? / be a new and different person? / be more or less the same?
- How long do you expect these changes to last?
- Is there anything about who _________ is that you wouldn’t be willing to lose/change in exchange for an improvement in their depression symptoms?

**4. Inter-personal relationships**

I now want to ask you about the impact DBS might have upon ________’s relationships with others, including yourself:

- Do you think your relationship with ________ will change after DBS?
  - If *yes*, how might it look like?
- In what ways would you like your relationship to change?
- Do you have any worries about how your relationship might change after DBS?
- Do you think __________’s relationships with other family and friends will change after DBS? If *yes*, how?

**5. Informed consent**

Like any surgical procedure, there are a number of risks and side effects associated with DBS:

- What risks or side effects *related to the surgery* are you aware of?
- What concerns do you have about the surgery?
- After surgery, what risks or side effects *related to the stimulation* are you aware of?
- What worries do you have about the stimulation?
- How informed do you feel you about the risks and side effects of DBS? In the short term (i.e. in undergoing surgery)? In the long-term?

**6. Stimulator management**

After the surgery, managing and controlling the stimulator becomes an important issue. __________’s stimulator settings are going to be adjusted throughout the trial.

- What changes do you expect could occur when these adjustments happen?
- How long do you think it will take for the changes to happen?
- Do you have any concerns about the stimulation adjustments and what changes might occur?
- Do you feel prepared to assist managing this?
- In terms of managing the stimulator yourself, have you thought about any issues you might face?
- *Prompt*: recharging, travelling with stimulator

We’re going to wrap up soon, I just have one final questions:

- Are there things that you would like to do or are looking forward to doing after DBS?
  - *Prompt*: socialising, leisure activities/hobbies, work/volunteering/study

**I’m just going to take a moment to look through my questions and make sure there are no points we’ve missed, while I’m doing this do you want to take a moment and think whether there are any issues important to you that we haven’t discussed?**

**CAREGIVER INTERVIEW SCHEDULE – POST-SURGERY**

**1. Background on DBS**

We met several months ago. We would like to better understand your experience since _________ received DBS.

- Can you explain what has happened since then? Has anything changed in your life? If so, please describe?
- Can you briefly comment on the experience of ______ having the DBS surgery itself?

**2. Experiences and perspectives**

- Do you recall what your original expectations regarding DBS? Were they met? (e.g., in terms of life activities and specific symptoms)?
- Have you seen any improvements in ________?
- Are there areas you have not seen improvements that you expected to?

**3. Personality and identity**

- Do you think having DBS has changed _______’s personality in anyway? If so, how?
- Do you feel like ________ is themselves since the surgery?
- Have your relatives and/or close friends noticed changes in _______? What kind of comments or observations have they made?
- What do you think brought on these changes (the device or feeling better)?
- Do you think _________ see themselves differently in anyway now?
- How have they adjusted to having the device as part of them and their body?

**4. Inter-personal relationships**

- Do you see yourself differently since ________ underwent DBS? When did these changes start to occur?
- Do you feel like you and _______ are different people now? How do you feel about the change?
- How would you describe your relationship since DBS?
- *Prompt:* better, easier, strained more complex?
- Are there tensions between you and ________ now related to the outcome of DBS?
- Are there tensions or conflicts that have resolved since DBS?
- Who do you seek advice from regarding any issues like these? Do you ever discuss these sorts of issues with your team at follow up appointments?
- How has ________’s relationships with others changed since having DBS?

**5. Informed consent and decision-making**

- Knowing what you know now, if you have your time again would you support ________ to undergo DBS?
- How informed about the risks and side effects of surgery do you think you were? Do you think you fully appreciated these?
- Are there things that have come as a surprise to you?
- Looking back, are there things you would have liked to have known?

**6. Device control issues**

- Who controls the stimulator being on/off, change the parameters, recharging? Has it brought about any challenges?
- Who do you contact if you have concerns about the stimulator?

**7. Public understanding and knowledge transfer**

- Are there things that the general public should know about DBS for depression?
- Have you or would you recommend this procedure to other patients/caregivers?

**Is there anything that we haven’t talked about that you would like to?**
