# Supplementary material for: Personal and relational changes following deep brain stimulation for treatment-resistant depression: A prospective qualitative study with patients and caregivers
Source: PLoS One. 2023 Apr 6;18(4):e0284160. doi: 10.1371/journal.pone.0284160 (PMC10079124; doi:10.1371/journal.pone.0284160)
Supplement: Attachment — Submitted filename: Response to Reviewers.docx [file pone.0284160.s002.docx]

Dear editor/reviewer,

Thank you for taking the time to review our manuscript and for your constructive feedback. Responses to reviewer comments (in bold) and other manuscript amendments are listed below. Page/line numbers align with the revised version of the manuscript containing track changes.

**Reviewer #1:** **This study qualitatively examined the personality, identity and interpersonal changes following DBS of bed nucleus of stria terminalis in 6 patients with treatment resistant depression. The authors devised a semi structured interviews focusing on i) background on DBS, ii) expectations/perspectives, iii) personality/identity, iv) interpersonal relationships, v) informed consent and decision making, vi) stimulator management. The interviews were conducted with participants and family care givers pre-DBS and 9 months post-DBS.**

**Previously published paper by the same authors titled “Nothing to lose, absolutely everything to gain” (Frontiers in Human Neuroscience Sep 2021) addressed two themes emerged from the questions on pre-DBS expectations/perspectives and post-DBS experiences and perspectives, pre- and post-DBS informed consent and decision making. The current paper focused on personality/ identity and interpersonal relationship.**

**Comments:
1. Although the results are presented in two parts in two papers, and mentioned as non-overlapping, there seem to be implicit overlap in conception, thematic analysis, and conclusions. Ideally these two papers should have been combined into one paper. Authors should clearly mention overlapping and non-overlapping components in methodology.**

The authors considered at length the option of consolidating all the findings from the study into a single paper. Due to the complex nature of the qualitative data collected (that includes multiple participant groups, timepoints, and research questions of interest), we concluded that attempting to condense all of these findings into a single paper would not be possible within the constraints of all leading clinical and scientific journals. Inclusion of all of the findings within a single paper would also not do justice to the data. We believe that the two papers addressed two different clusters of themes that are more or less relevant to different populations of readers. The currently published paper deals with questions of responsible research in DBS trials, and of primary interest to clinical and scientific teams conducting trials, whereas this current study examines the impact of DBS trials on personality and interpersonal relationship that would be of relevance to a range of disciplines (e.g., social science, philosophy), and not just clinicians and scientists. Including all themes within a single paper would make it overly dense and largely incomprehensible. In order to remain within journal word limits and allow for sufficient exploration of themes, the decision was made to publish two separate papers presenting two cohesive clusters of themes that pertain to different research and interview questions. We agree that greater clarity in needed to distinguish the overlapping and non-overlapping components of the two papers:

**EDIT TO MS [Pg. 5 Line. 101]:**

Separate data findings derived from these interviews, on the topics of informed consent, participant expectations and subjective patient outcomes, are reported elsewhere (27).

**2. Personality and identity are complex multifaceted psychological concepts. Participant’s response may be influenced by their level of understanding and knowledge about personality and identity. Did the authors brief the participants about the dimensions of personality and identity during the interview?**

We deliberately chose not to provide participants with a definition of personality or identity or any briefing on these concepts. We were not interested in their views on scientific or psychological constructs of personality or identify. Rather we were curious to see how participants made sense of these concepts for themselves, and how this influenced their perspectives on their experience of DBS and depression. This point has been included in the manuscript.

**EDIT TO MS [Pg. 6 Line. 129]:**

Pre-surgery interviews occurred 3 to 15-weeks prior to surgery (*M* = 46 minutes, range = 34-58 minutes) and explored participants’ knowledge of DBS and beliefs about how the patient’s personality, self and relationships may be affected (see Supplementary Material for interview schedules). Participants were not provided with a definition or description of ‘personality’. We were interested specifically in their personal experiences of personality and identity change. This was done to avoid restricting participants’ responses to a particular conceptual framework.

**3. Limitations of qualitative methods can be minimized by using mixed model. This need to be addressed in limitations**

**EDIT TO MS [Pg. 24 Line. 470]:**

While a mixed-method approach could have produced findings with both naturalistic and statistical-probabilistic generalisability, this approach was not feasible as the small experimental sample prohibited the conduct of robust quantitative analyses on psychometric data.

**4. Minor errors.
a) Introduction Page 3 line 66. “There have been no studies investigating patients experience of DBS in TRD”. This should be changed to there have been no in-depth studies investigating patient’s experience. There were two studies that examined patients subjective experience along with objective personality changes in NEO (Bewernick et al 2018 Psychol Med; Ramasubbu et al J 2021 Psychiatry Neurosci).**

This statement has been amended according to reviewer’s suggestion. The two studies have also been expanded upon in the discussion section.

**EDIT TO MS: [Pg. 3, Line. 69]**

While the efficacy and safety of DBS for TRD continues to be investigated via clinical trials, with mixed results (13), there have been no **in-depth** studies investigating patients’ experience of DBS and their perspectives on the psychosocial changes they undergo.

**EDIT TO MS: [Pg. 20, Line. 364]**

Two studies have investigated the impact of DBS for TRD on personality profiles using the NEO Five-Factor Inventory. Bewernick et al. (32) found no difference in Five-Factor scales at 6-months, 2 years or 5 years after DBS of the supero-lateral branch of the medial forebrain bundle (*n* = 21), despite patients obtaining an antidepressant response. The authors suggest this may reflect a ‘scar effect’ in which lengthy depressive episodes result in long-term, irreversible changes to personality. In contrast, Ramasubba et al. (31) found a significant decrease in Neuroticism and increase in Extraversion following DBS of the subcallosal cingulate (*n* = 22), with patients assessed regularly up to 15-months. The personality changes were associated with clinical improvement in depression, with the direction of these changes aligning with normative personality data. The contrasting outcomes across the two studies may be explained by differences in target nuclei, assessment timelines, and patient populations.

**b) Discussion: Page 21; line 380-381. “CBT has been recommended as a best practice component for DBS recovery and shown to augment treatment effects (43)”. This should be changed to “shown to augment treatment effects in obsessive compulsive disorder (43). Add on CBT has no effect on DBS recovery in treatment resistant depression (TRD) (Ramasubbu et al 2020 Lancet psychiatry).**

**EDIT TO MS: [Pg. 22, Line. 410]**

In OCD samples, cognitive behavioural therapy (CBT) has been shown to augment DBS treatment effects (43) and is recommended for best-practice post-operative care (44). In TRD samples, there is no evidence indicating CBT augments the DBS treatment effect (45); however, psychological therapy is likely to play an important role in relapse prevention.

**Reference list**

**The following references have been edited to reflect updated details. We are unaware of any included citations that have been retracted.**

**References with updated information**

20.	Thomson CJ. The Neuroethics Blog. [2021 Feb 23]. Available from: http://www.theneuroethicsblog.com/2021/02/ill-wear-this-as-badge-of-honour.html.

23.	Mosley PE, Robinson K, Coyne T, Silburn P, Breakspear M, Carter A. ‘Woe Betides Anybody Who Tries to Turn me Down.’ A Qualitative Analysis of Neuropsychiatric Symptoms Following Subthalamic Deep Brain Stimulation for Parkinson’s Disease. Neuroethics. 2021;14:47-63.

27.	Thomson CJ, Segrave RA, Fitzgerald PB, Richardson KE, Racine E, Carter A. “Nothing to Lose, Absolutely Everything to Gain”: Patient and Caregiver Expectations and Subjective Outcomes of Deep Brain Stimulation for Treatment-Resistant Depression. Front Hum Neurosci. 2021;Deep Brain Stimulation Think Tank: Updates in Neurotechnology and Neuromodulation, Volume II:1-13.

31.	Ramasubbu R, McAusland L, Chopra S, Clark DL, Bewernick BH, Kiss ZHT. Personality changes with subcallosal cingulate deep brain stimulation in patients with treatment-resistant depression. J Psychiatry Neurosci. 2021;46(4):E490-E499.

36.	Thomson CJ, Segrave RA, Carter A. Changes in Personality Associated with Deep Brain Stimulation: a Qualitative Evaluation of Clinician Perspectives. Neuroethics. 2021;14:109-24.

49.	Bell E, Maxwell B, McAndrews MP, Sadikot A, Racine E. A review of social and relational aspects of deep brain stimulation in Parkinson's disease informed by healthcare provider experiences. Parkinsons Disorder. 2011;2011:1-8.

50.	Flores Alves Dos Santos J, Tezenas du Montcel S, Gargiulo M, Behar C, Montel S, Hergueta T, et al. Tackling psychosocial maladjustment in Parkinson's disease patients following subthalamic deep-brain stimulation: A randomised clinical trial. PLoS One. 2017;12(4):1-15.

51.	Mosley PE, Robinson K, Dissanayaka NN, Coyne T, Silburn P, Marsh R, et al. A Pilot Trial of Cognitive Behavioral Therapy for Caregivers After Deep Brain Stimulation for Parkinson's Disease. J Geriatr Psychiatry Neurol. 2020;34(5):454-65.

52.	Slatman J, Widdershoven G. An Ethics of Embodiment: The Body as Object and Subject. In: Meacham D, editor. Medicine and Society, New Perspectives in Continental Philosophy. Philosophy and Medicine. 2015. p. 87-104.

54.	Jakobs M, Helmers AK, Synowitz M, Slotty PJ, Anthofer JM, Schlaier JR, et al. A multicenter, open-label, controlled trial on acceptance, convenience, and complications of rechargeable internal pulse generators for deep brain stimulation: the Multi Recharge Trial. J Neurosurg. 2019:1-9.

**New references added to manuscript**

32.	Bewernick BH, Kilian HM, Schmidt K, Reinfeldt RE, Kayser S, Coenen VA, et al. Deep brain stimulation of the supero-lateral branch of the medial forebrain bundle does not lead to changes in personality in patients suffering from severe depression. Psychol Med. 2018:1-9.

35.	Zuk P, Sanchez CE, Kostick-Quenet K, Munoz KA, Kalwani L, Lavingia R, et al. Researcher Views on Changes in Personality, Mood, and Behavior in Next-Generation Deep Brain Stimulation. AJOB Neurosci. 2022:1-13.

46.	Acevedo N, Castle D, Groves C, Bosanac P, Mosley PE, Rossell S. Clinical recommendations for the care of people with treatment-refractory obsessive-compulsive disorder when undergoing deep brain stimulation. Aust N Z J Psychiatry. 2022:1-7.

47.	Ramasubbu R, Clark DL, Golding S, Dobson KS, Mackie A, Haffenden A, et al. Long versus short pulse width subcallosal cingulate stimulation for treatment-resistant depression: a randomised, double-blind, crossover trial. The Lancet Psychiatry. 2020;7(1):29-40.
