# Supplementary material for: Personal and relational changes following deep brain stimulation for treatment-resistant depression: A prospective qualitative study with patients and caregivers
Source: PLoS One. 2023 Apr 6;18(4):e0284160. doi: 10.1371/journal.pone.0284160 (PMC10079124; doi:10.1371/journal.pone.0284160)
Supplement: Attachment — Submitted filename: Response to Reviewers.docx [file pone.0284160.s003.docx]

Dear editor/reviewers,

Thank you for taking the time to review our manuscript and for your constructive feedback. Responses to reviewer comments (in bold) and other manuscript amendments are listed below. Page/line numbers align with the revised version of the manuscript containing track changes. Underlined sections reflect new additions to the manuscript.

**Reviewer #2: Thomson et al. conducted semi-structural interviews to 6 treatment-resistant depression (TRD) patients who underwent BNST deep brain stimulation (DBS) and 5 caregivers pre- and post-operatively. The interview topics were related to self-concept, device acceptability, and relationships with caregivers, which were generally overlooked by physicians, and current clinical score (such as HAMD and BDI) do not present these themes. This qualitative study explores in-depth understanding of relationship between alleviation of depression with above experience of DBS for TRD, and provide new insight into a more comprehensive antidepressant effect evaluation of DBS for TRD. As a clinician who is heavily involved in such procedure, I enjoy reading the manuscript, and find it illuminating. Below are some comments I have:

Major:

1. Regarding the candidate who completed pre but not postop interview: this may be a source of bias since the clinical effect may be less satisfactory than those who chose to complete both.**

The candidate who did not complete the postop interview had an unsatisfactory clinical response to DBS and this contributed to them not completing the interview (the severity of their depressive illness was extreme). While the absence of their voice in the postop data may bias these findings (although the concept of bias is viewed somewhat differently in qualitative research), the inclusion of their caregiver allowed for some of their postop experiences (which were generally negative) to be incorporated into the findings.  **2. No mentioning of clinical efficacy in terms of alleviation in depressive symptoms (HAMD or BDI). I’m curious about change in QoL, too.**

Semi-structured interviews and clinical measures were conducted at a similar timepoint (9-months post-DBS), but were not precisely time locked (conducted on the same day). Given this and patients’ fluctuations in response, we have not commented on this within the paper. Long-term clinical outcome data will be presented in the efficacy study publication (footnote inserted mentioning this). But broadly speaking, the clinical outcomes measures collected at the 9-month timepoint reflected participants’ subjective perceptions. Those who reported little-to-no benefit (*n* = 3), or slight worsening of depression (*n* = 1) were non-responders according to MADRS and HAM-D and maintained a ‘severe’ depression status according to BDI. For one patient who reported a subjectively meaningful improvement, this was reflected in both clinician and self-rated measures (MADRS = response (>50% decrease), HAM-D = close to response (45% decrease), BDI = from ‘severe’ to ‘mild’). However, for the other patient who reported a meaningful improvement, self-rated measures reflected their subjective reports (BDI = ‘severe’ to ‘minimal’), but clinician-rated measures did not (MADRS = no response, HAM-D = no response). While the patient experienced distinct improvements in mood and interest, functional recovery was slow and the process of social reintegration challenging. This may account for some of the discrepancy in subjective and objective (clinician-rated) outcomes. Regarding quality of life, according to the Q-LES-Q-SF, QoL scores remained stable for all, with the exception of the one responder whose score increased from 4% to 68%, and a non-responder whose score decreased from 21% to 7%.

**EDIT TO MS [Pg. 5, Footnote]**

Clinical trial details, including inclusions/exclusion criteria, participant characteristics, surgical information, and full psychometric outcomes, will be presented in a forthcoming publication. Any correspondence regarding the clinical trial should be directed to paul.fitzgerald@anu.edu.au. **3. Line 189-192: How do you define subjectively meaningful vs more modest antidepressant effects?**

Clarifying descriptors added to manuscript.

**EDITS TO MS [Pg. 10, Line. 208]**

With DBS, patients’ perspectives of themselves were greatly influenced by the procedure’s perceived benefit. Those who experienced a subjectively meaningful antidepressant benefit (i.e., described a pronounced improvement in mood that was sustained over multiple months) conveyed a sense of restored self (*n* = 2). Those who experienced modest or transient benefits (*n* = 3) (i.e., described subtle improvements in mood and/or notable lifts lasting only 2-3 days duration) recognised encouraging moments of their prior self (e.g., increased curiosity, interest in work activities). **4. Line 196: Any measurable correlation between decreased depression and transformation in self concept? I understand that the authors are trying to conduct a qualitative study, but as a reader from a neurosurgeon/neuroscientist background, I can’t help but to try to capture some correlations between the two.**

My observation based off this small sample is that there was a correlation between decreasing depression and transforming self-concept, with those experiencing more pronounced antidepressant benefit reporting a greater sense of transformation (or restoration) of self. In terms of a measurable correlation, this is outside the aim and scope of this qualitative study.

**5. In the current format, the data is laid out in a relatively mixed fashion. A summary table of patients’ demographic, pre and post depression rating scale score, and personal and relational changes may make it easier for the reader to see the big picture.**

A table has been inserted on page 5 that provides an overview of participant demographics. Our reasoning for not including pre and post outcome measures in addressed above (response 2). While we agree it would be helpful to have a table providing full case summaries, we are conscious of protecting participants’ confidentiality. Presenting individual participant characteristics (as opposed to group demographics) alongside qualitative excerpts runs the risk of making individual participants and their comments identifiable (potentially to their spouse, parent, child etc.). We explored alternative methods for laying out the results section; however, the current layout was the best approach for ensuring that the timepoints were distinct (pre/post DBS) and allowing sufficient space for both patient and caregiver experiences to be examined across the three themes.

**6. Line 337: What is the medication/psychotherapy protocol in this trial? Do patients continue to receive best medical treatment during trial period?**

The line the above comment is referring to is regarding caregivers’ use of medication/psychotherapy. ‘Caregivers’ has been inserted to provide clarity.

Edits have been made to the Procedure section regarding patient therapies during the trial.

**EDIT TO MS [Pg. 6, Line. 144]**

Any existing treatments (medications/psychotherapy) were kept constant during the blinded phase with participants able to make changes during the open-label follow-up.

**Minor

1. Line 53: “microelectrode” is misleading. This is the probe we use in the OR to record spike signals before inserting DBS leads.**

Corrected. **2. Line 106: female/male ratio is 5:1. What kind of impact will it have on the study?**

Edits have been made to the Limitations section acknowledging that this ratio impacts the transferability of the findings. We are reluctant to make assumptions about how this would have impacted the nature of the findings themselves.

**EDIT TO MS [Pg. 26, Line. 577]**

The majority of the patient sample were female (5 of 6), which may reflect the higher representation of females in depressed populations. This gender imbalance is likely to have influenced the nature of the observed findings, potentially reducing the transferability of these males with depression. **3. What is the SD/SEM of age/duration of illness?**

This information has been included in Table 1 [Page 5.] **4. Line 109: The candidate who chose not to complete postop interview is a patient or caregiver?**

The candidate was a patient. Edits made to clarify this.

**EDIT MS [Pg. 5, Line. 114]**

One DBS candidate who completed a preoperative interview did not complete a postoperative interview as it was deemed too burdensome for the patient. **5. Line 112: SD/SEM would help.**

Added to Table 1 [Page 5.] **6. Line 119: Does the location of the interview (home vs research center) or method (in person vs phone vs video conference) have any effect on interview results?**

It is possible that the different locations and methods could have affected the interviews and results (e.g., increased anxiety for interviewee in a clinical setting, less rapport developed with interviewer via video conference, absence of visual cues for interviewer over the phone). While it would have been preferable to have consistency in interviewing procedures, logistically this was not possible and our focus was on making participation as simple as possible for participants (recognising that the participation requirements of the concurrent clinical efficacy trial were considerable).  **7. Line 246: Which IPG (model type) is used in this study?**

**EDIT TO MS [Pg. 6, Line. 139]**

Participants then underwent DBS surgery and were implanted with Medtronic Activa 3389 electrodes in the BNST. **8. Line 377: Any measurable correlation between personality restoration and degree of benefit? Is DBS restoring personality that leads to antidepressant effect, or the antidepressant effect brings back the personality, or simultaneously? This is a challenging question, as there is not enough data to support any theory, but would like to know what the authors think.**

See above comments to response 4 regarding measurable correlations. On the matter of the directional relationship between depressive symptoms and personality; we suspect that it is the alleviation of the depressive symptoms that allows their personality to emerge, that while depression is severe and sustained, their personality is masked or inhibited.

**9. Line 411: Does the time of the relationship between patients and caregivers have any impact on how DBS changes relationships?**

Details about the relationship length have been included in Table 1 [Page 5.]. All had very long-term relationships (range 24 – 50 years). Relationship time did not appear to impact how DBS changed relationships. If the above comment was in fact about relationship ‘type’ rather than ‘time’, some further comments have been included in the manuscript regarding this.

**EDIT TO MS [Pg. 24, Line. 507]**

In De Haan and colleagues’ (52) OCD sample, patients reported that both they and their partners had to “get used to” the new them. As OCD had always been present in their relationship, couples had created lives that worked around the illness. For the married couples in the current sample (*n* = 4), their relationships were established well before the onset of depression. Therefore, partners were experiencing varying degrees of ‘reacquaintance’ with their loved one (depending on benefit gained from DBS). Regardless of outcome, DBS created challenges for marital relationships. These couples, however, had weathered similar storms across their long-term, committed relationships. So too was the case for the parent-child pair.  **10. Recharging is a common cause of patient frustration. As a clinician, I would like to know specifically which part(s) of recharging (length, frequency, method, etc) is most annoying, and how to make it more tolerable (e.g., recharging time reduced from 15 to 5 minutes each day?)**

The following comment in the results section speaks to the specific parts of the recharging process that were most annoying for patients:

“Frustration was associated with difficulty establishing a reliable connection, inconsistency in how long and often recharging was required, recharging taking longer than originally expected, and the growing need to prioritise recharging in daily life.” [Page. 13, Line 207]

Regarding how the process of recharging could be improved or made more tolerable, some suggestions have been added to the discussion (underlined sections below). Please note, specific targets (e.g., reducing time from 2 hours to 1) are not included as this was not explored directly with participants.

**EDIT TO MS [Pg. 23, Line. 485]**

The recharging needs for individuals with psychiatric conditions typically differs from those with movement disorders (higher voltages resulting in faster battery depletion), as does their psychological and cognitive profiles. In OCD, post-operative education has been shown to assist with device-related anxiety (55), but further investigation is needed. Notable memory difficulties, which the majority of current patients reported (*n* = 4), can affect retention of device-related information. For this reason, perioperative device education may require modification for TRD purposes. This may include consideration of when device education is offered (e.g., not immediately after surgery) and conducting multiple sessions. Those who had received support from their device company representative in the months following surgery had found this helpful for improving their recharging process (achieving a more reliable recharging connection). A reduction in how long and how often recharging is required would increase the tolerability of the process. This is dependent on the capacity of the current technology. As DBS for psychiatric indications progresses, so too should device technology and research evaluations of patients’ device-related needs (17).

**11. Any chance the authors can share the interview recordings and/or the transcript? Of course anonymization is required, but this would help us conduct similar research in the future, too.**

The sharing of interview recordings/complete transcript data (including de-identified) with any person outside of the research team was not a condition participants agreed to when consenting to take part in the study. Participants only agreed to have relevant excerpts of their transcript used as required within the manuscript. Given the particularly sensitive nature of the data (e.g., participants discussing intimate details of their relationship with the patient/caregiver) we are particularly conscious of maintaining participants’ confidentiality. Even after the process of de-identification, it could be possible to establish a participant’s identity from the full transcript data. We do, however, recognise the value of this rich data for other interested researchers and will include this condition in future studies.

**Reviewer #3: Thomson and colleagues report on the lived experiences of 6 patients with treatment-resistant depression (TRD) and 5 caregivers before and after DBS. The paper documents the views on self-concept, device acceptability, and relationships before and after DBS. The manuscript I am reviewing seems to be a revision after at least one previous peer review. It might be good to note that I am seeing this manuscript for the first time. This manuscript is a well-written, interesting paper and the used qualitative method gives a broader view on these topics than standardized instruments. Therefore, I think this paper contains new and valuable information, and is a valuable addition to the literature. I have only minor comments that might improve this paper.

1. The results section is filled mostly with words like ‘some’, ‘many’ and ‘others’ without specifying the number of patients or caregivers that named this particular theme. Could the authors specify the number throughout the manuscript?**

We acknowledge that an indicator of frequency as suggested would be helpful; however, we wish to avoid extensive quantification within the results. This is an argument made by many qualitative researchers, in part because each participant interview is unique, and the questions asked within them (beyond the interview guide) can differ. Quantification suggests only a certain number had a particular experience, when in fact others may have also. The nature of that interview and the questions asked may not have led to it being discussed. We provide an editorial reference on this issue from Professor Joanne Neale and colleagues in the journal *Addiction* (Prof Neale is the Associate Editor for Qualitative Research at *Addiction*).

Neale, J., Miller, P., & West, R. (2014). Reporting quantitative information in qualitative research: Guidance for authors and reviewers. *Addiction*, *109*, 175-176.

Some semi-quantification with non-specific terms is used e.g., “a few”, “some”, “many” to indicate frequency in a relative sense. Single cases are remarked upon but are typically presented in a way that does not exclude others from potentially having had the same experience. We have provided explicit frequencies when reporting types of personality change, our reason being that each participant was asked the same specific questions on this topic to prompt these responses. We have also provided explicit frequencies for participants’ perceived benefit from DBS, as we have found this to be a particular point of interest for readers and reviewers in the past, who request these frequencies specifically.

**2. Although the discussion contains an interesting section on personality and identity in treatment-resistant depression, it is very general and missing a link with the presented data in the Results. For instance, what do the data in this paper tell us about and relate to the data found on the NEO-FFI? And what can these data tell us about the improbability of personality restoration? How are the perceived changes in personality similar to or different from those reported by Parkinson or OCD patients?**

We agree that parts of this section are too general and require linking back to the study findings. This particular sub-section (Personality and Identity in Treatment Resistant Depression) has been re-ordered and edited in order to make these link between the broader literature and the current findings clearer.

**3. Minor detail: the structure in the results is 1) personality; 2) device acceptability and 3) relationships. In the discussion device acceptability and relationships are discussed in a different order. I think it would be more systematic to adhere to the same order in the discussion as used in the results.**

As suggested, the discussion sections have been re-ordered to align with the themes in the results.

**4. I spotted a typo on p.20 (Discussion): ‘Ramasubba’ should be ‘Ramasubbu’.**

Corrected.
