# Supplementary material for: Personal and relational changes following deep brain stimulation for treatment-resistant depression: A prospective qualitative study with patients and caregivers
Source: PLoS One. 2023 Apr 6;18(4):e0284160. doi: 10.1371/journal.pone.0284160 (PMC10079124; doi:10.1371/journal.pone.0284160)
Supplement: Attachment — Submitted filename: Response to Reviewers.docx [file pone.0284160.s004.docx]

Dear Academic Editor

Thank you for reviewing our revised submission. Below is our response to your recommendation for our manuscript.

**The presence of quantitative results is little bit confusing to the reader. It seems that the study is a mixed-method not a qualitative one. I do suggest describe the participants' characteristics without offering quantitative data.**

Additional participant information in the form of a table containing quantitative participant demographic data was introduced following recommendations from a reviewer. Prior to this, we had described participant characteristics within text, using minimal quantitative data. Given your above recommendation, we have decided to revert to our previous approach. Table 1 has been removed, with details provided within the ‘Participants’ text.
